# Supplementary material for: Effect of clinical isolate or cleavage site mutations in the SARS-CoV-2 spike protein on protein stability, cleavage, and cell–cell fusion
Source: J Biol Chem. 2021 Jun 20;297(1):100902. doi: 10.1016/j.jbc.2021.100902 (PMC8214756; doi:10.1016/j.jbc.2021.100902)
Supplement: Supplemental Figures S1–S3 [file mmc1.pdf]

Supporting Information

Effect of clinical isolate or cleavage site mutations in the SARS-CoV-2 spike protein on protein stability, cleavage, and cell–cell fusion

Chelsea T. Barrett[1], Hadley E. Neal[1], Kearstin Edmonds[1], Carole L. Moncman[1], Rachel Thompson[1], Jean M. Branttie[1], Kerri Beth Boggs[1], Cheng-Yu Wu[1], Daisy W. Leung[2], and Rebecca E. Dutch[1]*

[1]Department of Molecular and Cellular Biochemistry, University of Kentucky, Lexington, Kentucky, USA

[2]Division of Infectious Diseases, Department of Medicine, Washington University School of Medicine in St Louis, St Louis, Missouri, USA

Materials included:

- Three supporting figures

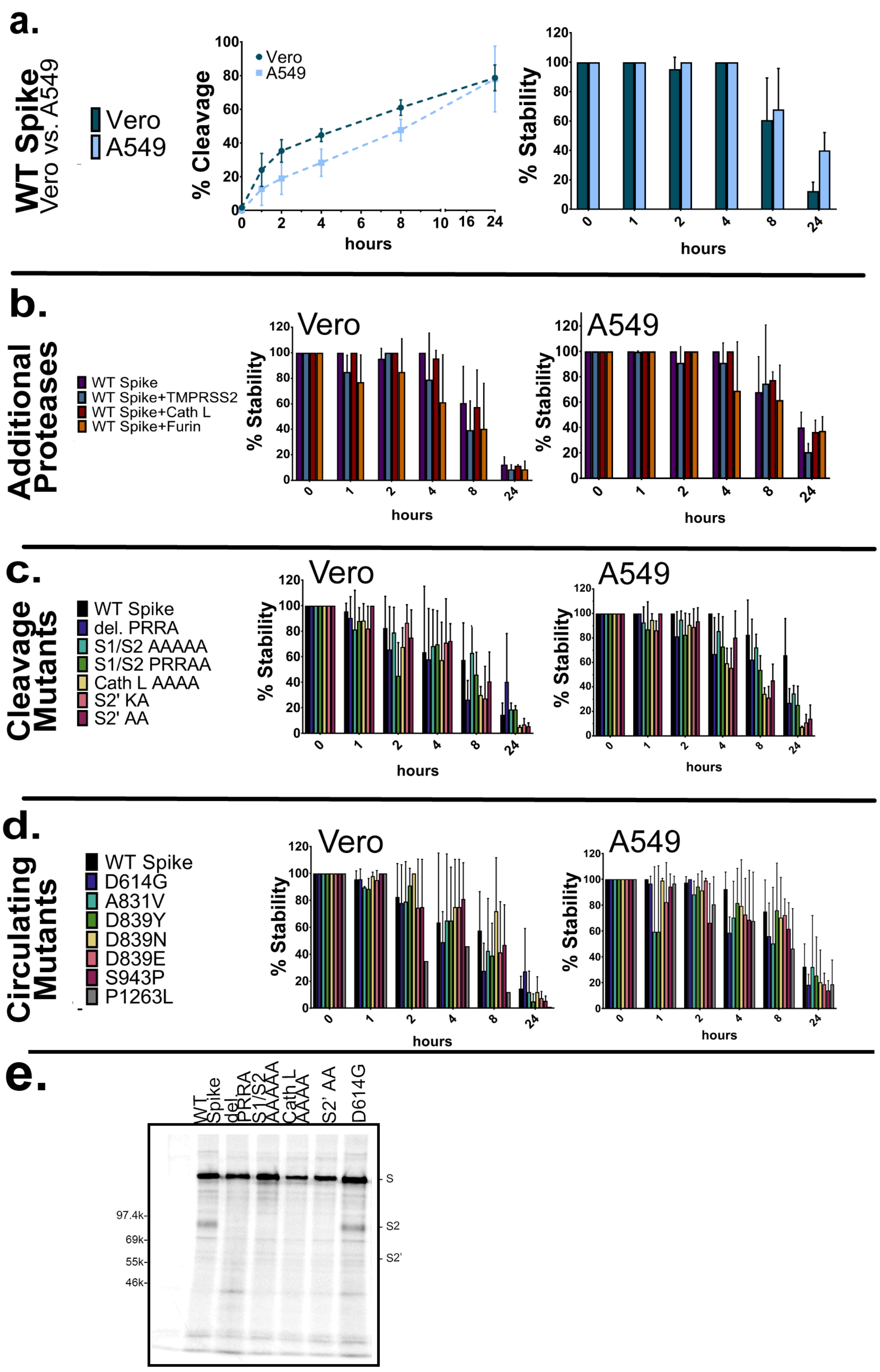
a.
WT Spike
Vero vs. A549
Vero
A549
% Cleavage
hours
% Stability
hours
b.
Additional Proteases
WT Spike
WT Spike+TMPRSS2
WT Spike+Cath L
WT Spike+Furin
Vero
A549
% Stability
hours
c.
Cleavage Mutants
WT Spike
del. PRRA
S1/S2 AAAAA
S1/S2 PRRAA
Cath L AAAA
S2' KA
S2' AA
Vero
A549
% Stability
hours
d.
Circulating Mutants
WT Spike
D614G
A831V
D839Y
D839N
D839E
S943P
P1263L
Vero
A549
% Stability
hours
e.
WT Spike
del. PRRA
S1/S2 AAAA
Cath L AAAA
S2' AA
D614G
97.4k
69k
55k
46k
S
S2
S2'

Figure S1: SARS-CoV-2 protein stability comparisons. A) Wt S was transfected into Vero or A549 cells, metabolically labeled for one hour, and chased for times indicated. Blots (shown in Fig 1a) were quantified using band densitometry. Both percent cleavage and percent stability are shown. B) Overall protein stability at each time point from blots in Fig. 1D were quantified. C) Overall protein stability at each time point from blots in Fig. 3B were quantified. D) Overall protein stability at each time point from blots in Fig. 4B were quantified. E) Vero cells expressing wt S or the indicted mutants were metabolically labeled for 5 hours. The predicted position of S2 and S2' are indicated, based on the molecular weight.

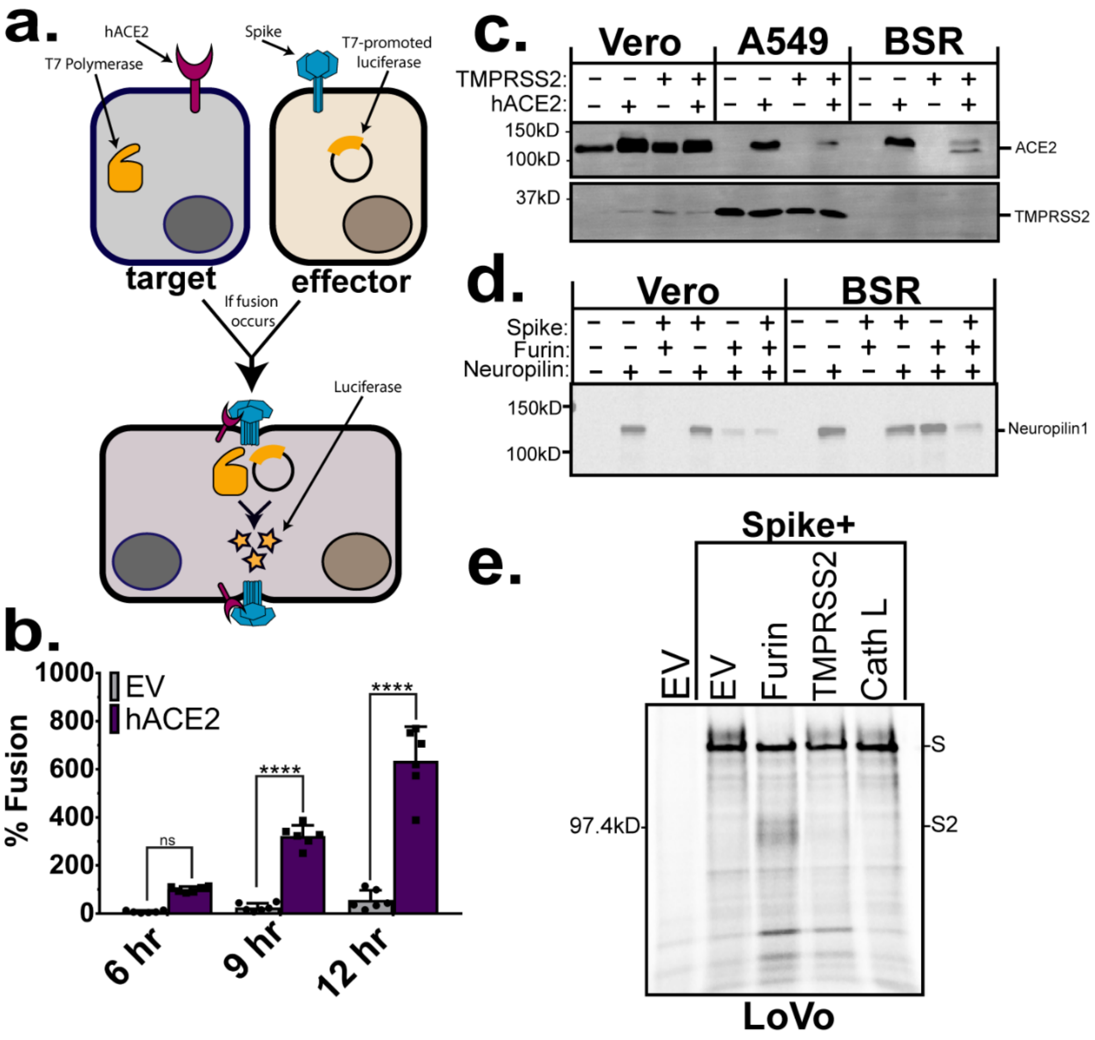

Figure S2: SARS-CoV-2 Spike Luciferase Reporter Gene Assay Controls. a) The experimental schematic for the luciferase reporter gene fusion assay used throughout the manuscript is shown here. In all experiments, the target cells used are BSR/T7 cells that constitutively express a T7 polymerase. Effector cells used are Vero cells in most experiments and A549 cells in Figure 2c. b) Various overlay times were tested in a luciferase reporter gene assay, effector cells (Vero) were transfected with wt S, and target cells were transfected with empty vector (EV) or hACE2. Results represent three independent experiments, each performed in duplicate. Significance was determined by two-way ANOVA, ****; $p<0.001$. c) Endogenous or transfection levels of TMPRSS2 and hACE2 were assessed in Vero, A549, and BSR/T7 cells by western blot. d) Transfected expression levels of Neuropilin in the experimental conditions from Fig 2d. e) LoVo cells transiently expressing S alone, or S with the indicated protease were metabolically labeled for 6 hours. Results are representative of four independent experiments.

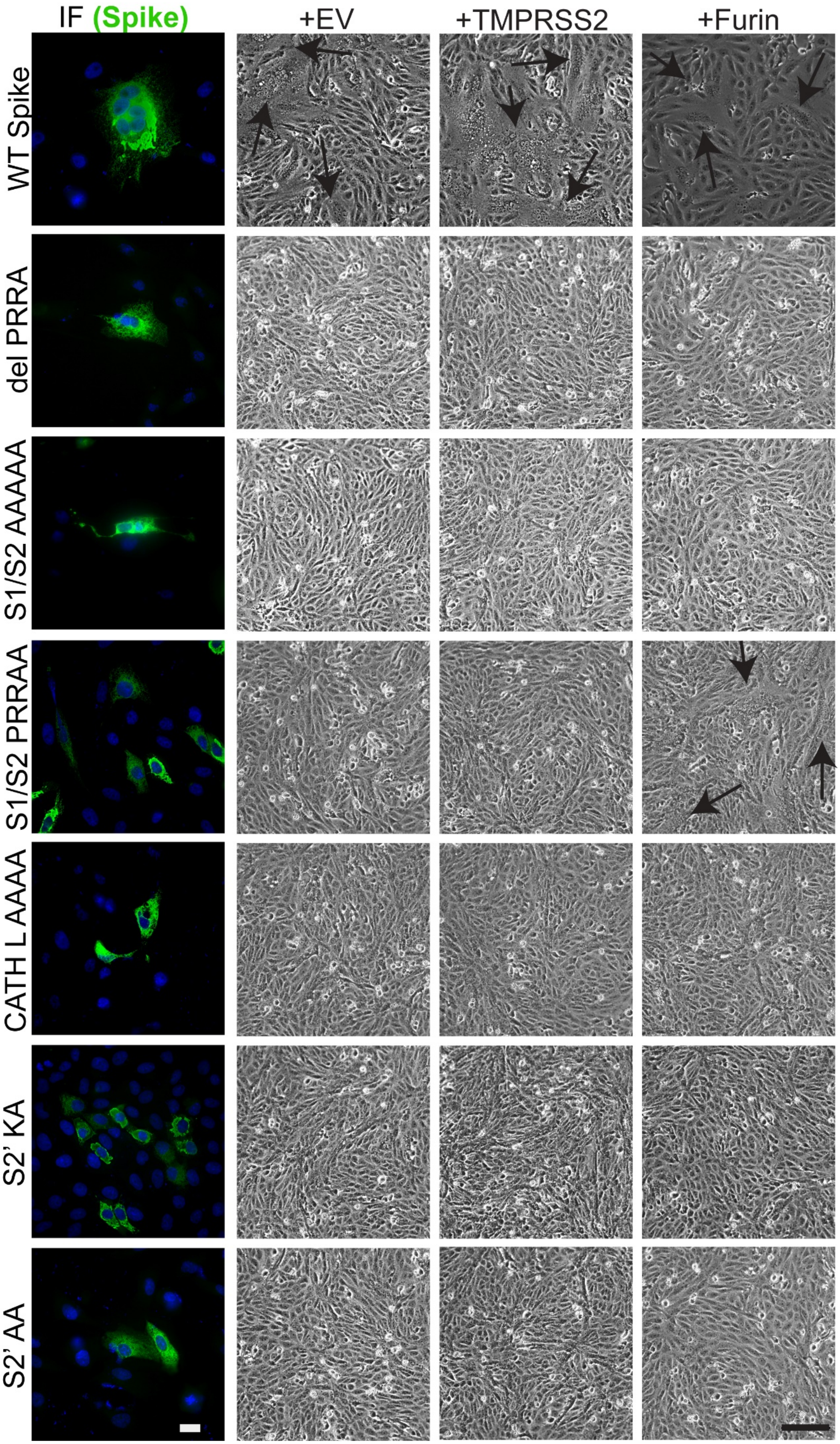
IF (Spike)
+EV
+TMPRSS2
+Furin
WT Spike
del PRRA
S1/S2 AAAAA
S1/S2 PRRAA
CATH L AAAA
S2' KA
S2' AA

Figure S3: Mutations made at any of the three potential cleavage sites abolish syncytia formation. Vero cells were transfected with wt S or each of the cleavage mutants. The first panel shows immunofluorescence of wt S or S cleavage mutants (green, magnification bar is 20µm). The remaining panels show syncytia formation at 24 hours post transfection with S or mutants co-expressed with EV, TMPRSS2, or furin. Syncytia are indicated by the black arrows, magnification bar is 100µM.
